# Parents and Vivisection

**Published:** 1913-05-17

**Authors:** Arthur Westerman

**Affiliations:** London.


### The Editor's Letter-Box.

### Parents and Vivisection.

*To the Editor of* The Hospital.

Sir,—Having seen your comments about my giving boric acid to a baby, may I write that, believing that a very small dose of pure boric acid could not be harmful to a healthy child, and knowing that preserved cream is very largely used by all classes, I made my observations with a sincere desire to find out if this acid produced any signs of gastric irritation. If it can be proved that it does so, the Local Government Board should not allow it to be added to cream, which is such a valuable adjunct to the food of infants.

I have taken boric acid myself in its pure form, and also in preserved cream, without any ill-effect; in the summer months I would rather use preserved cream.

The amount taken by the baby was about the same as she would have taken if she had been given preserved cream containing not more than ½ per cent. preservative. There was no sign of indigestion during a period of six days, and during four days the weight increased ¼ lb.

Although the press had a misleading account of my evidence, I know now that I did wrong in allowing an interview at all, and I had no idea it would be used in the way it was.

Arthur Westerman, M.D.

London.

